# Impact of Ultrasound Pretreatment on Juice Yield and Bioactive Content in Juice Produced from Selected Berries Fruit

**DOI:** 10.3390/foods13081231

**Published:** 2024-04-17

**Authors:** Jan Piecko, Monika Mieszczakowska-Frąc, Karolina Celejewska, Justyna Szwejda-Grzybowska

**Affiliations:** Fruit and Vegetable Storage and Processing Department, The National Institute of Horticultural Research, Konstytucji 3 Maja 1/3, 96-100 Skierniewice, Poland; monika.frac@inhort.pl (M.M.-F.); karolina.celejewska@inhort.pl (K.C.); justyna.grzybowska@inhort.pl (J.S.-G.)

**Keywords:** ultrasound, strawberry, blackcurrant, raspberry, juice

## Abstract

The aim of the work was to investigate the effect of ultrasound application during juice pressing. The impact on pressing yield and extraction of bioactive compounds during production of juice from strawberry, blackcurrant and raspberry was evaluated. Juice pressing was carried out after four kinds of mash pretreatment. The tested objects were heated to 55 °C, treated by ultrasound, and/or macerated with an enzyme. The obtained juices were analyzed for the content of bioactive compounds and compared to the ‘Control’ sample, which was the juice pressed without any pretreatment. Although the results presented here do not conclusively show that enzymatic maceration can be completely replaced by the use of ultrasound, in the case of strawberry and raspberry fruit, juice yield after ultrasound treatment increased almost as much as after enzymatic maceration. Additionally, in the case of raspberry fruit, the antioxidant activity of the juice samples was definitely improved when ultrasound was used. The results from this experiment also showed that it is possible to separate the effect of temperature from the effect of mechanical and chemical actions in ultrasound treatment.

## 1. Introduction

In addition to apples, of which Poland is one of the world’s largest producers, currants (6.0%), strawberries, and raspberries (6.0%) account for a significant share of total fruit production. Additionally, Poland is one of the largest producers of berry fruits in Europe [1]. In 2017, the combined yield of currants (blackcurrant and redcurrant), strawberries, and raspberries exceeded 400,000 tons, which accounted for 85% of the total berry fruit yield in Poland [2]. In detail, strawberry fruit accounted for 34%, blackcurrant fruit for 31%, while raspberry fruit accounted for 20% of the total crop production in 2018 [3]. The large scale of production translates into a large base of raw material available for processors, who are eager to use it. In some seasons, 68% of the strawberry harvest and as much as 78% of the raspberry harvest was allocated for processing [4]. Blackcurrant fruits are rarely consumed in their fresh form, but more often in liquid form, blended with other fruit juice, nectars, or other products, so it can be said that the entire harvest is directed for processing.

Berries are widely known for their positive effects on human health. Due to their low calorie (below 70 kcal), low fat (less than 0.5 g/100 g) [5], high dietary fiber content, presence of micronutrients and composition of phytochemicals—in particular anthocyanidins and vitamin C—berries occupy a very important place in the human diet. For example, blackcurrants have shown significant therapeutic potential in a number of conditions, confirmed by evidence from clinical trials. A review of the beneficial and therapeutic effects of blackcurrant consumption involves its effect on the brain, lungs, bones, kidneys, heart, and skin [6]. A similar feature applies to strawberries, the most widely consumed berry in Europe. Strawberries in clinical trials are considered in terms of their antioxidant, cardioprotective, anti-inflammatory, antihyperglycemic, antiobesity, anticancer, antimicrobial, and neurodegenerative effects on human body [7]. Raspberries also contain a number of polyphenolic compounds with antioxidant properties, which play a significant role in reducing the risk of diseases, including chronic diseases, by mitigating the harmful effects of oxidative stress [8].

Sound waves at frequencies above 20 kHz are called ultrasound, and exhibit all the characteristic properties of sound waves except for the human ear’s ability to perceive them. Depending on the frequency, ultrasound is divided into three categories: power ultrasound (20–100 kHz), high-frequency ultrasound (100 kHz–1 MHz) and diagnostic ultrasound (1–500 MHz). Ultrasounds in the range of frequency from 20 to 100 kHz have the ability to induce the phenomenon of cavitation, which involves the formation of gas bubbles. During the bubbles implosion, physical effects (microstreaming, microstreamers, microjets, and shock waves) and chemical reactions (sonolysis of water molecules and reactive oxygen species formation) can occur [9,10]. There are a large number of current and potential applications of ultrasound in food processing, the most common being cooking, freezing, drying, pickling, degassing, filtration, defoaming, emulsification, oxidation, and cutting [11]. The main advantage of using ultrasound-based technology is that it reduces the duration of the process, and improves the efficiency and hygiene of the process [12]. The use of ultrasound as an additional tool to inactivate microorganisms and enzymes in the preservation process is particularly beneficial [9,13]. It has also been shown that ultrasound has a synergistic effect on pectin hydrolyzing enzymes by modifying both the substrate structure and the enzyme itself, leading to more efficient pectin degradation [14], which is beneficial in juice production. Higher activities of pectin lyase and polygalacturonase after ultrasound application were observed, whereas in the case of pectin methylesterase, this ultrasound effect was not noticed. The degradation of pectin in the fruit mash before pressing is, in many cases, an unavoidable step to increase the efficiency of the process. On the other hand, pectin degradation products are released into the juice, increasing the fiber content and stabilizing anthocyanins in juice, which is also a desirable process in terms of the dietary value of the product [13]. Another potential direction of application is the ultrasound-assisted extraction of antioxidants from herbs or by-products of food production. Operating parameters of the devices used in the research work as ultrasonic amplitude, sonication time, extraction temperature and solvent-to-solid ratio are selected as crucial parameters for extraction optimization [15].

Despite numerous studies documenting great potential of ultrasound, there is no strong trend to replace traditional methods in favor of the ultrasound application, and compared to other emerging technologies, it is not the most frequently used [16]. It is pointed out that the main reason for such a situation is a poor understanding of ultrasound-based techniques and a commitment to tradition [12].

Keeping in mind the large scale of berry fruit production in Poland and the enormous health-promoting potential of blackcurrant, raspberry and strawberry, research aimed at increasing the transfer of bioactive compounds into juice seems meaningful. Ultrasound, considered a sustainable and environmentally friendly technology, is an interesting tool to achieve such a purpose. The research shows that, in selected cases, the use of technology based on the ultrasound treatment of mash before pressing the juice can replace enzymatic maceration, which shortens the process and can offer economic savings. Nowadays, it is a trend to produce fruit juices without using an enzymatic pre-treatment to maintain the highest possible level of fiber in the final product. However, depending on the kind of fruit, it can be difficult or even impossible to squeeze juice and omit enzymatic maceration. One of the possible solutions can be incorporating ultrasound into this process. The aim of this study was to compare the pressing efficiency and the content of bioactive compounds in juices produced with the ultrasound treatment of raw material from selected berries. Although ultrasound used in food processing is considered as a non-thermal method, the action of a high intensity sound waves causes an increase in temperature, which is sometimes overlooked. With the aim of separating the effect of temperature and ultrasound, an additional sample was included in the experiment where fruit pulp was heated to the same temperature that the ultrasound generated after 10 min of exposure (55 °C-as the optimum temperature for the enzyme). The conducted experiment brings new data to the possibility of using ultrasound as a substitute for enzymatic maceration or as a complement to it in the case of raspberries, strawberries, and blackcurrant juice.

## 2. Materials and Methods

### 2.1. Plant Material

The fruit used in the study came from commercial cultivation and were purchased during the 2020 harvest season from local producers in central Poland. The experiment was conducted on 3 fruit species: strawberries (*Fragaria x ananassa* var. ‘Florence’), red raspberries (*Rubus idaeus* var. ‘Polka’), and blackcurrants (*Ribes nigrum* var. ‘Tihope’). Immediately after being transported, the fruits were washed, sorted, and the stalks or other debris were removed and then cooled down to 2 °C. After that, the fruits were frozen and stored at −25 °C until processing. 

### 2.2. Mash Treatment and Juice Pressing

**Figure 1:**
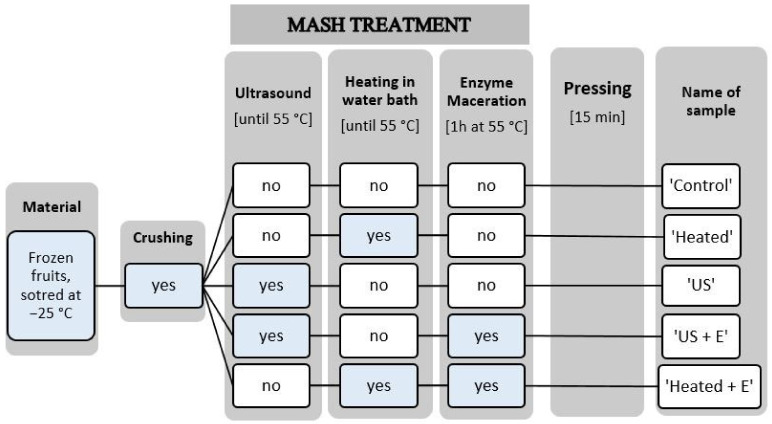
Scheme of the experiment.

The scheme of this experiment is presented in Figure 1. For each treatment, 200 g of fruit was crushed in a laboratory blender (Thermomix, Vorwerk, Wuppertal, Germany), after which fruit pulp was transferred to a glass beaker (250 mL). The ‘Control’ sample was pressed immediately. To consider the effect of temperature on juice quality, a ‘Heated’ sample was included in experiment. The ‘Heated’ sample was pressed after heating in the laboratory bath to 55 °C (an optimal temperature for the enzyme activity). The ‘US’ sample was pressed after sonication. The samples were treated in an ultrasonic homogenizer at a frequency of 20 KHz and 750 W power (Cole-Palmer Instrumental Company, VCX-750, Vernon Hills, IL, USA) with a 13 mm diameter probe. The ultrasonic processor operated with a constant amplitude of 80%. Ultrasonic processing was carried out for approximately 10 min and was terminated when the fruit pulp reached a temperature of 55 °C. During ultrasound treatment, fruit pulp was stirred to equalize ultrasound action throughout the beaker. This treatment was carried out by submerging the probe in a 250 mL glass beaker (height 9.5 cm, diameter 7.0 cm) and 200 mL of fruit mash was placed inside the beaker. The tip of the probe was submerged to half the depth of the beaker filling (3.0 cm). The ‘US + E’ sample was sonicated (ultrasound treatment procedure described above) and after enzyme addition, incubated in 55 °C for 1 h in a laboratory bath. The ‘Heated + E’ sample was pressed after mash heating in a laboratory bath to 55 °C, enzyme addition, and incubating in 55 °C for 1 h in a laboratory bath. When ‘US + E’ and ‘Heated + E’ samples are compared, the influence of ultrasound on enzymatic macerated fruit mash is considered. Different enzymes were used regarding the different fruit species. For strawberries and raspberries it was Pectinex^®^ Ultra Passover (Novozymes, Glendale, CA, USA) and in the case of blackcurrants Pectinex^®^ Ultra Color (Novozymes). The enzymatic treatment of the fruit was carried out with the chosen enzyme at a dose of 50 g/t. All samples were pressed for 15 min using the Instron 4303 texture analyzer, equipped with a special cylindrical container for juice pressing. The obtained juice was heated to 90 °C before chemical analysis in order to cut enzymatic reactions and microbiological spoilage. The experiment was conducted in three repetitions. 

The pressing yield of the juice was calculated according to equation:

(1) $$yield\;[\%]=\frac{weight\;of\;juice\;[g]}{weight\;of\;mash\;[g]}\times 100$$

### 2.3. Chemical Analysis

#### 2.3.1. Soluble Solids Content

The soluble solids content (°Bx) of juice samples was determined using the refractometric method and was carried out in three independent replicates (Mettler-Toledo, refractometer type RE50, Schwerzenbach, Switzerland). The device is equipped with a system to stabilize the temperature of the sample during measurement to 20 °C.

#### 2.3.2. HPLC Analysis of Polyphenols

A HPLC analysis of the phenolic compounds was performed according to the method described by Nielsen et al. [17] with some modifications using an Agilent 1200 series HPLC (Hewlett-Packard, Palo Alto, CA, USA) system equipped with a DAD detector. Separation was performed using a Phenomenex^®^Fusion RP column (250 mm × 4.6 mm; particle size 4 µm). The elution conditions were as follows: 1 mL min^−1^, temperature 25 °C, wavelength: 280 nm (flavan-3-ols), 320 nm (phenolic acids), 360 nm (flavonol) and 520 nm (anthocyanins); mobile phase consisting of water: formic acid (95:5 (*v*/*v*)) (A), and acetonitrile (B) in gradient flow. The calculations of polyphenols were quantified by calibration with the standards of individual polyphenols. The polyphenol content was expressed in mg/100 mL of juice.

#### 2.3.3. HPLC Analysis of L-Ascorbic, Malic and Citric Acid

The L-ascorbic, malic, and citric acid contents were determined using high-performance liquid chromatography (Agilent 1200 HPLC system, equipped with a DAD detector) using Supelcosil LC-18 column (250 mm × 4.6 mm; 5 µm) with a precolumn according to IFU procedures. A 1% phosphate-buffered solution KH_2_PO_4_, pH 2.5 was used as the mobile phase. The isocratic flow was 0.8 mL min^−1^, at a temperature of 30 °C. The detection of L-ascorbic acid was performed by absorbance at 244 nm and 210 nm for malic and citric acid. The samples were homogenized in 6% HPO_3_, and filtered through filter paper. The acids were quantified using a calibration curve for L-ascorbic, malic, and citric acids. The results were expressed as mg/100 mL of juice.

#### 2.3.4. Antioxidant Activity

The antioxidant activity was determined according to Re et al. [18]. Free-radical scavenging activity was determined using the ABTS•+ radical cation. The sample (500 μL) was mixed with an ABTS•+ solution (5 mL). Next, the reaction was carried out for 6 min, and the mixture was kept at room temperature in a dark place. Absorbance was measured at 734 nm using a spectrophotometer—the Cary 3E UV-Visible (Varian). At least four measurements were made for each sample at different concentrations to reduce the initial ABTS•+ solution absorbance from 20% to 80%. The linear regression method was applied to calculate the content of the sample, leading to a 50% decrease in the ABTS•+ solution absorbance, and recalculated to Trolox equivalents as mg per 100 mL of juice. The extract was prepared as follows: 10 mL of juice was mixed with 20 mL of 70% methanol and was then extracted for 10 min in an ultrasonic bath. The suspension was filtered through a Whatman No. 3 filter paper.

### 2.4. Statistical Analysis

The statistical analysis was performed with the use of STATISTICA 13 (Dell Inc., Tulsa, OK, USA). The HSD Tukey’s test was selected for the comparison of the quality parameters of juices. The analysis of treatments ran as a completely randomized design and was conducted with one-way ANOVA. The means that comparison was performed at a significance level of *p* < 0.05.

## 3. Results and Discussion

### 3.1. Pressing Yield

**Figure 2:**
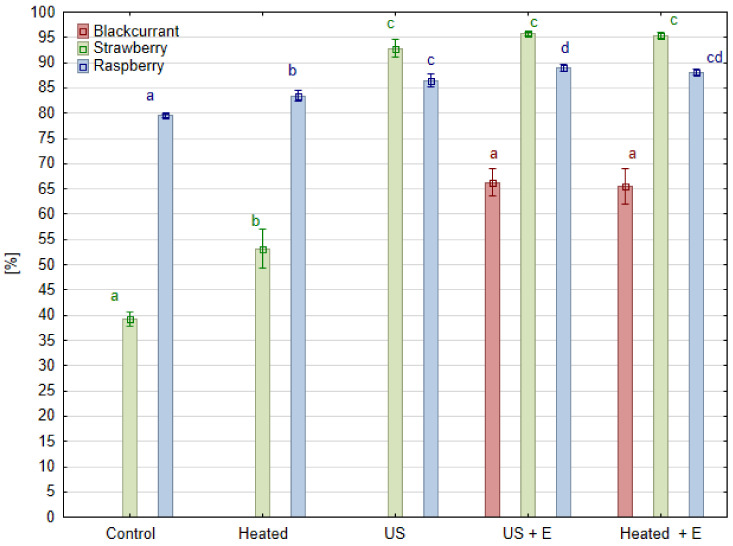
Juice yield (%) in different kinds of raspberry, strawberry, and blackcurrant mash treatment before pressing. Means, for one species, marked with the same letter in the same color do not differ significantly (*p* = 0.05) according to Tukey’s test (n = 3).

Juice pressing yield is a parameter which reflects how much juice was obtained in relation to the amount of raw material taken for pressing. From the juice producers point of view, this is the most important parameter because the amount of juice obtained translates into the profitability of production. Each species included in the experiment behaved differently in terms of the effect of ultrasound treatment. In the case of raspberry fruit, the yield obtained in the ‘Control’ and ‘Heated’ juice samples gained 79.5% and 83.4%, respectively (Figure 2). Hence, the baseline pressing yield without any treatment was high, and heating the mash before pressing increased the pressing yield by only 3.9% compared to the ‘Control’. The use of enzymatic maceration of raspberry mash (‘Heated + E’ sample) resulted in a 8.6% increase in the pressing yield, while when the mash was treated with ultrasound before maceration (‘US + E’ sample), a 9.6% increase was obtained compared to the ‘Control’. Such results are in agreement with work comparing juice pressing efficiency, where the control juices from raspberry fruits (without maceration, heated to 50 °C) reached 75.0% yield. When enzymatic maceration was used, a yield of 84.9% was obtained [19]. When the juice was pressed immediately after ultrasound treatment ‘US’, the increase in pressing yield was comparable to the effect of enzyme maceration ‘Heated + E’, and increased by 5.8% compared to the ‘Control’. Better results was observed in mulberry fruit as the juice yield after ultrasonic treatment increased by 27.0% compared to juice without mash treatment [20]. In the case of strawberry fruit, the observed results were quite different. In the case of the ‘Control’ and ‘Heated’ fruits, the pressing yields were low, and reached only 39.2% and 53.5%, respectively. Heating the strawberry mash caused a significant increase (by 14.3%) in the efficiency of the pressing process compared to the ‘Control’. The use of enzyme maceration of strawberry mash ‘Heated + E’ caused a 56.5% increase in the yield. While the mash was treated with ultrasound before maceration ‘US + E’, a 53.5% increase was achieved when compared to the ‘Control’. When the juice was pressed immediately after ultrasound treatment ‘US’, the increase in pressing yield was comparable to the effect of using enzyme maceration ‘Heated + E’ and amounted to 56.2% compared to the ‘Control’, and by 41.9% compared to the ‘Heated’ mash sample. In this case, ultrasonic treatment has equal efficacy in pectin degradation to that carried out with the enzymatic maceration of commercial products designed to treat berry mash before juice pressing. Sonication treatment replaced the effect of enzymatic maceration and enabled the juice to be pressed with profitable yield.

Blackcurrant mash proved to be a much more difficult material to press and it was not possible to perform it after the ‘Control’ and ‘Heated’ pretreatment. This is in line with practice as enzyme preparations have been used in the juice processing industry since the 1930s [21]. During blackcurrant juice production, pectinolytic enzymes are indispensable for degrading pectins, which are released during mashing from the cell walls. In the case of blackberry fruit, similarly low yields were obtained by other authors when, before pressing, the mash was not subjected to enzymatic maceration. In one of these cases, the ‘Control’ juices (without maceration) had a low pressing yield of 37.6%. When maceration was used, juice yields varied, depending on the type of treatment before pressing, from 70.6% when the pulp was macerated immediately after heating to 50 °C and 70.4% when the mash was heated to 85 °C before maceration [19]. Even lower pressing yields were obtained in the experiment conducted on an industrial scale, where not subjecting the mash to enzymatic maceration allowed for obtaining only 31.0% of juice yield [22]. In our experimental design, heating the blackcurrant mash was not enough to make juice pressing possible. Treating the mash only with ultrasound also was not a sufficient processing method to allow for obtaining any amount of juice. The use of an enzyme maceration ‘Heated + E’ resulted in a pressing yield of 66.1%, while when the blackcurrant mash was treated with ultrasound before maceration ‘US + E’, a pressing yield of 67.7% was reached. Hence, in the case of blackcurrant, ultrasound treatment itself had no effect on the yield of juice. A similar effect on juice yield was obtained by other authors testing the ultrasound treatment in a laboratory bath over 5, 10, and 15 min. Regardless of the currant variety (black, red or white), the effect of the ultrasound treatment was insignificant [23]. In line with this statement, ultrasound treatment, enzymatic maceration, or heating showed no effect on juice yield of the *Berberis amurensis* Rupr. fruit mash [24]. However, from a study on similarly difficult to obtain banana juice, combining ultrasound with enzymatic maceration enabled a 17.3% higher yield in comparison to enzymatic maceration itself [25]. The increase in pressing efficiency after ultrasound mash treatment may be related to the reduction in viscosity caused by pectin degradation. Model studies of pectin solution indicate that ultrasonic treatment can reduce the viscosity of the solution to a level comparable to the result of enzymatic maceration. This phenomenon is explained by a reduction in the number of agglomerates and changes in the particle structure, rather than a reduction in particle size, so the authors postulated that this treatment did not replace enzymatic maceration, but emphasized the synergistic effect of ultrasound and the activity of the pectin hydrolyzing enzyme [14].

### 3.2. Soluble Solids

The content of soluble solids is presented in Table 1. There were no statistical differences in the content of soluble solids in raspberry and blackcurrant juices, regardless of the kind of pretreatment. An increase in soluble solids was observed in strawberry juice after using a combination of ultrasound with enzymatic maceration ‘US + E’. The 4.0% increase, compared to the untreated ‘Control’, was presumably mainly due to the heating of fruit mash. Ultrasound applied without enzyme maceration had no effect, while the use of enzyme maceration (without ultrasound) increased soluble solid content by 3%, as did heating to 55 °C without ultrasound. Similar observations were noted on blueberry, blackcurrant, and raspberry fruit when the mash was heated to 50 °C and enzymatic maceration at this temperature was compared. The soluble solid content increased slightly for all three species [19]. Similar trends of total soluble solids content were observed, which ranged from 11.44 °Brix (for Control) to 11.95 °Brix when mulberry fruit mash was treated in an ultrasonic bath for 60 min [20]. In the case of banana mash treated with ultrasound, a significant increase in soluble solid content was shown only when the treatment was combined with enzymatic maceration [25].

Although ultrasound has great potential to improve extraction [9] it is not always applicable to the mash treatment process in juice production. In one example, in which three varieties of blackcurrant were used for the experiment, despite the fact that a small change was observed, the effect of ultrasound on the mash showed no trend [23]. Also, with regard to the effect of ultrasonic treatment of the juice, the impact on this parameter is not clear. In an experiment in which orange juice treated with different ultrasonic amplitude levels was used, no changes were shown in soluble solid content compared to the untreated ‘Control’ [26]. Consistent with this, treating grape juice with ultrasound, despite the long exposure time (90 min), did not change the content of the soluble solids [27]. On the other hand, treating carrot juice with ultrasound for 60 min allowed for the observation of an increase in the solubility of the sugar, and thus, an increase in soluble solid content [28]. 

### 3.3. Phenolic Compounds

**Figure 3:**
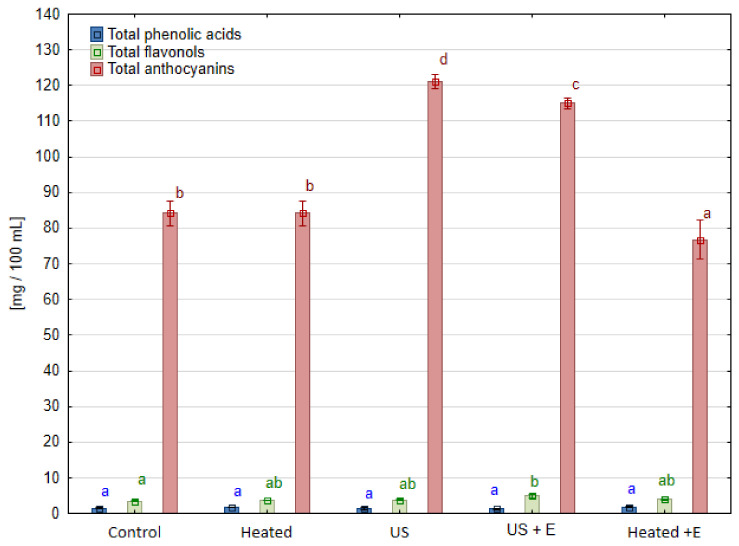
Phenolic compounds groups in raspberry juice obtained using different mash treatment. Means, for each phenolic group, marked with the same letter in the same color do not differ significantly (*p* = 0.05) according to Tukey’s test (n = 3).

**Figure 4:**
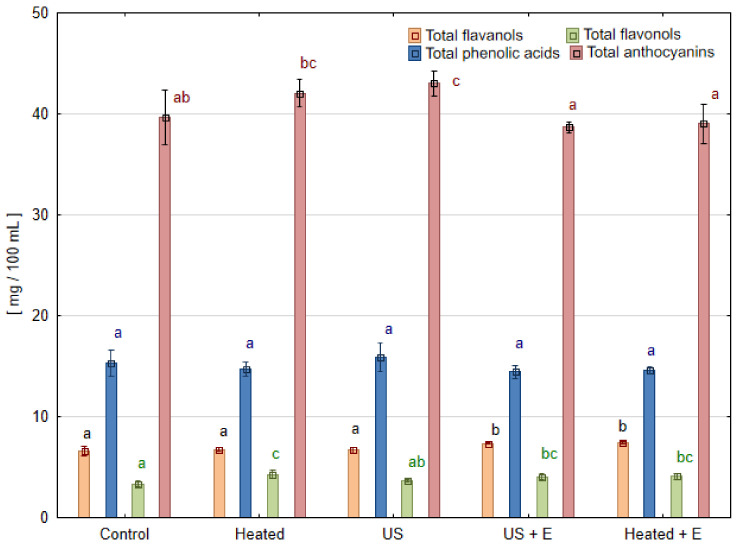
Phenolic compound groups in strawberry juice obtained after different mash treatment. Means, for each phenolic group, marked with the same letter in the same color do not differ significantly (*p* = 0.05) according to Tukey’s test (n = 3).

**Figure 5:**
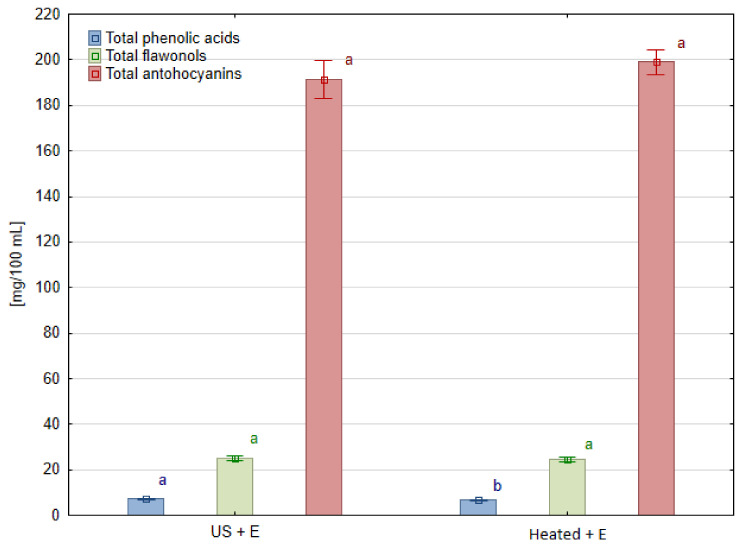
Phenolic compound groups in blackcurrant juice pressed after different mash treatment. Means, for each phenolic group, marked with the same letter in the same color do not differ significantly (*p* = 0.05) according to Tukey’s test (n = 3).

The contents of the main classes of phenolic compounds for all samples tested are presented in Figure 3, Figure 4 and Figure 5. In raspberry juice (Figure 3), the three classes of phenolic compounds were detected: phenolic acids, flavonols, and anthocyanins; in strawberry juice (Figure 4) four classes were marked: flavanols, phenolic acids, flavonols, and anthocyanins; in the case of blackcurrant juice (Figure 5) phenolic acids, flavonols, and anthocyanins were determined. Amongst the group of flavonoids, the most abundant subgroup found in berries fruit are anthocyanins, which dye their skin in intense colors. The results showed the positive effects of ultrasound application on this subgroup for raspberry juice, while no such relationship was observed for strawberry and blackcurrant juice. Table 2 shows the content of these compounds measured using the HPLC method in raspberry juice. In raspberry fruits treated with ultrasound before pressing ‘US’, and before enzyme maceration ‘US + E’, the content of these compounds increased compared to the untreated ‘Control’ by 40.3% and 34.2%, respectively. Anthocyanins in raspberry juice demonstrated interesting behavior upon ultrasound mash treatment. The content of cyanidin 3-sophoroside was more than ten times higher, while the content of cyanidin 3-rutinoside and cyanidin 3-glucosyl-rutinoside was almost ten times lower. Heating the mash without sonication did not affect the content of phenolic compounds, while enzymatic maceration slightly reduced their content compared to the ‘Control’. In this case, an effect of ultrasound was observed here, with no significant effect of temperature itself. Based on the results of this analysis, the content of cyanidin-3-sophoroside and cyanidin-3-glucoside increased after ultrasound treatment (‘US’ and ‘US + E’ samples) while cyanidin-3-glucosyl-rutinoside and cyanidin 3-rutinoside were negatively affected by ultrasound treatment. The content of cyanidin-3-sophoroside, compared to the ’Control’ samples of raspberry juice, increased by 91.3% when ultrasound was applied before maceration ‘US + E’, while it increased by 102.9% when juice was pressed immediately after US treatment. In the case of cyanidin-3-glucoside, the content increased by 35% in ‘US + E’ sample, while it increased by 40.9% for ‘US’ juice. The different level of retention of individual anthocyanins is related to the fact that the stability of compound is influenced by its chemical structure [29].

The phenomenon observed in our research indicates the influence of US on the structure of the anthocyanin molecule. We hypothesize that ultrasound waves cause the rhamnose molecule to disconnect from cyanidin-3-rutinoside, transforming it into cyanidin-3-glucoside. However, in the case of the anthocyanin cyanidin-3-glucosyl-rutinoside, which contains two sugars: glucose and rutinose (α-L-rhamnosyl-glucoside), under the influence of US, rhamnose is also disjoined. In this way, anthocyanin containing two glucose molecules, i.e., sophoroside disaccharide, is formed, which is observed as increasing the content of sophoroside cyanidin.

Moreover, the stability of anthocyanins in juice depends on many factors: temperature, the pH of the medium, access to light, the presence of oxygen, ascorbic acid, sugars, metal ions and enzyme activity [30]. Differences in anthocyanin stability were found depending on the medium or storage and processing conditions. For example, heating pomegranate juice at 90 °C for 5 h resulted in 76–87% loss of total anthocyanin [31], while when blueberry juice was heated to 100 °C, 32% anthocyanin degradation was observed after 20 min [32].

A significant impact of ultrasonic waves was also observed in the case of the content of ellagic acid in the tested raspberry juices (Table 2). Juices obtained from fruits treated with ultrasound (‘US + E’ and ‘US’) had 26–30% more ellagic acid than the ‘Control’ sample. The opposite effect was observed under heating alone (‘Heated’ sample), where the ellagic acid content was lower than in the control sample, about 10%.

Due to the higher content of pelargonidin-3-glucoside, cyanidin-3-glucoside, cyanidin 3-malonylglucoside, and pelargonidin 3-(6″-malonylglucoside) in strawberry juices pressed immediately after US treatment ‘US’ (Table 3), the total content of anthocyanins was higher compared to the ‘Control’ (Figure 4). The juice sample pressed immediately after ultrasound treatment (‘US’) showed a 19.7% increase in cyanidin-3-glucoside content and a 14.9% increase in pelargonidin-3-(6″-malonylglucoside) content when compared to the ‘Control’. However, the content of these compounds in the ‘US’ sample was not significantly different from juice pressed after heating the ‘Heated’ sample, so it can be concluded that it was the temperature that mattered in the extraction of these compounds. Pelargonidin-3-glucoside, as the most abundantly present anthocyanins in strawberry juice, turned out to be stable regardless of ultrasound treatment or enzymatic maceration. The opposite behavior was seen in that of cyanidin-3-glucoside, which increased in all samples compared to the ‘Control’. Pelargonidin-3-(6″-malonylglucoside) retention was different, increasing only in samples without maceration while ultrasound treatment had no effect on its content compared to the ‘Control’.

Studying these results, it is possible to track down a slightly positive effect of the application of ultrasound on quercetin derivatives (A, B, C), but it is not enough to be significant for the total content of flavonols. What is more, the concentration of these compounds in the juice was very low (3–5 mg/100 mL). A much greater effect was observed in the study where the anthocyanin content of blackberry juice was investigated. In the samples produced with enzymatic maceration, up to 64.0% more of these compounds were recorded than in juice obtained without maceration [22].

The contents of phenolic compounds in blackcurrant juices measured using the HPLC method are shown in Table 4. In the case of this fruit species, only methods of treatment containing enzymatic maceration allowed us to obtain juice samples, so the results concern only two experimental combinations: ‘US + E’ and ‘Heated + E’. Particular anthocyanins, regardless of the treatment method used, were not statistically different, except for delphinidin 3-glucoside. Its content was lower in juice obtained after ultrasound treatment ‘US + E’ than in heated one ‘Heated + E’. However, the total content of anthocyanins and flavonols did not differ (Figure 5). Overlooking the insignificant changes in most of the detected phenolic compounds, it can be noted that by replacing the heating of blackcurrant fruit mash with ultrasound treatment, 8.3% more quercetin-3-glucoside can be extracted into juice. Also, a 6.5% increase in the content of phenolic acids (chlorogenic acid derivatives) was observed in the ‘US + E’ juice compared to ‘Heated + E’ treatment.

Differences in the content of individual compounds after ultrasound treatment are also reported by other authors. For example, in a study where sonication was carried out in a laboratory bath, the total phenolic content increased by 8.0% after 5 min and 16.0% after 10 min of blackcurrant mash exposure. However, in the same study, anthocyanins content showed no change after ultrasound treatment [23]. The opposite results were obtained in the case of *Berberis amurensis* juice, where the total anthocyanin content increased by 67.3% when the mash was treated with ultrasound, compared to the juice obtained from the untreated sample. Specifically, an increase in anthocyanin content was associated with the content of peonidin-3-glucoside. However, the total content of phenolic compounds from the untreated mash was equal to that of the juice from the ultrasonically treated mash, while heating or enzymatic maceration of mash led to a 25% increase [24]. 

Very interesting results were obtained by using ultrasound in aqueous extraction from the solid parts of the fruit. Authors have provided evidence that, in the extraction of polyphenols from blackcurrant seeds and pomace, there is no need to use organic solvents when ultrasound was applied [21]. In an aqueous extraction solution, the propagation of ultrasound is much easier, and, therefore, the uniformity of the effect improves significantly. This phenomenon may be the reason for the lack of effectiveness in the treatment on dense substances such as blackcurrant fruit mash. It has also been noted that the content of some polyphenols can increase even when the juice is treated after squeezing, when the extraction efficiency of these compounds from the raw material is unrelated. This phenomenon is explained by the release of cell wall-bound compounds that degrade during ultrasound-induced cavitation or the attachment of hydroxyl groups to phenolic rings [27].

### 3.4. Ascorbic, Malic, and Citric Acid

Table 5 shows the content of ascorbic, malic, and citric acids in the tested juices of the three berry species. Malic and citric acids are the main organic acids found in berry fruits [33]. Blackcurrant juices had the highest ascorbic acid content among the juices tested with an average value of 129 mg/100 mL. This is only slightly more than the content indicated by other authors studying ascorbic acid content in currant juices [23]. The ultrasound action during mash pretreatment did not affect the ascorbic acid content in the blackcurrant juice as it is shown in Table 5. A similar situation was observed in strawberry juice. The average content of ascorbic acid was 29.8 mg/100 mL, and individual treatments did not manifest any effect on ascorbic acid concentration. Raspberry juice contained the lowest amount of ascorbic acid among the samples tested, with an average value of 22.0 mg of ascorbic acid per 100 mL. At the same time, the type of mash treatment had the greatest impact on its content among tested fruit species. In the ‘Control’ juice sample, the ascorbic acid content was higher than in the other samples except from the ‘Heated’ juice where it was not statistically different. Ultrasound treatment ‘US’, as well as combined with maceration ‘US + E’ resulted in a slight reduction in ascorbic acid content. Maceration, in turn, (‘Heated +E’) resulted in the highest vitamin C decrease (17.2%) compared to the ‘Control’. Ultrasound treatment ‘US’, as well as enzymatic maceration ‘Heated + E’, has a similar negative impact on ascorbic acid, decreasing its content by 14.2 and 14.9%, respectively. 

A similar result was obtained by the authors comparing juice made with or without enzymatic maceration. In this case, maceration resulted in a 12.0% reduction in ascorbic acid content [22]. Several studies, however, indicated the positive effect of the ultrasound treatment on ascorbic acid content in juice. It is likely that this observation is due to the reduction in oxygen which can oxidize ascorbic acid. For example, vitamin C content of grapefruit juice significantly increased in a sonicated juice sample, compared to the untreated ‘Control’. A 90 min ultrasound treatment of the juice resulted in an almost 30% increase in ascorbic acid content [27]. In this case, the only reason explaining this result may have been the inhibition of degradation. In another study, where white currant mash was treated by ultrasound for 5 min, an increase in the content of vitamin C by 58% was recorded [23]. An even stronger effect was observed in mulberry juice, where the ascorbic acid content after 60 min of ultrasonic treatment increased by 73%, compared to juice obtained without mash treatment [20]. However, the effect of ultrasound mash treatment on the ascorbic acid content in fruit juices is not yet clear, and the results are inconsistent. For example, a study conducted on strawberry mash found a 20% decrease in ascorbic acid content in the juice after mash treatment with ultrasound in 50 °C [34]. An interesting look at the ascorbic acid content of the juices tested is their ability to meet the recommended daily intake (RDI). Because each juice has a different content of this vitamin, it should be considered separately. The RDI for ascorbic acid for people living around the world varies significantly depending on state regulations and ranges from 40 to 200 mg/day [35]. In the European Union, the RDI for ascorbic acid is different for each gender: 95 for women and 110 mg/day for man [36]. Considering the average RDI for both genders (102.5 mg/day), about 470 mL of raspberry juice, 345 mL of strawberry juice, and only 80 mL of blackcurrant juice must be consumed daily to meet the RDI for ascorbic acid.

Blackcurrant juices appeared to contain the highest amount of malic and citric acid of average values of 386 and 4700 mg/100 mL, respectively, compared to strawberry and raspberry juice (Table 5). No effect of ultrasound treatment of mash before maceration on the content of the abovementioned acids was observed. Equally, in the case of strawberry juice, no effect of ultrasound application on the content of any of these acids was observed. Enzyme maceration has a positive impact on citric acid content in strawberry juice, an 11.5% increase was recorded for the ‘US + E’ sample and 10.7% for ‘Heated + E’. The opposite behavior was observed for malic acid in raspberry. In comparison to the ‘Control’, juices that were obtained using maceration of the pulp before pressing ‘US + E’ and ‘Heated + E’ had 5.2 and 7.7% lower contents of malic acid, respectively.

### 3.5. Antioxidant Activity

**Figure 6:**
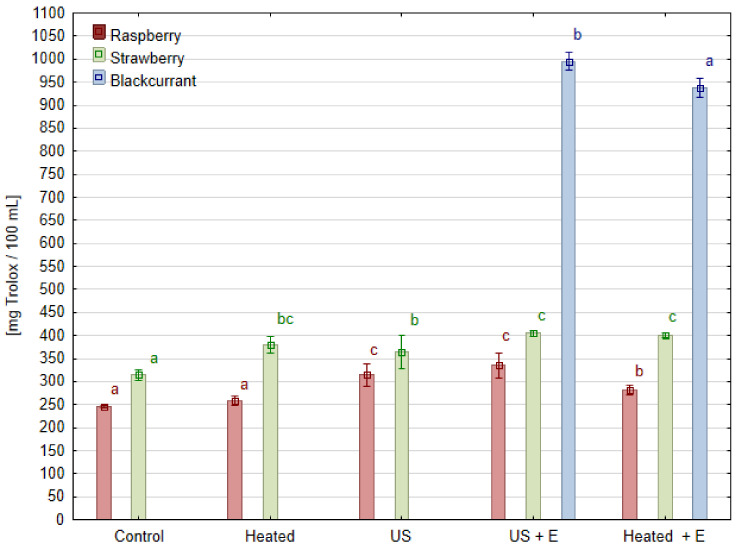
Antioxidant activity of juice obtained after different mash treatment. Means, for each species, marked with the same letter in the same color do not differ significantly (*p* = 0.05) according to Tukey’s test (n = 3).

The antioxidant activity of the juices measured by the ABTS test is shown in Figure 6. Its value ranged from 286.9 for raspberry juice to 966.5 [mg Trolox/100 mL] for blackcurrant juice due to its high anthocyanins and ascorbic acid content. Raspberry juice, with the lowest antioxidant activity among the juices tested, simultaneously showed the strongest tendency for the positive effects of ultrasonic treatment. The use of enzymatic maceration resulted in a 14.5% increase in antioxidant activity, while when the raspberry mash was treated with ultrasound before maceration ‘US + E’, a 36.6% increase in antioxidant activity was obtained compared to the ‘Control’. Juice produced immediately after ultrasonic treatment (‘US’) has a higher antioxidant activity than the ‘Control’ by 28.0%. Evidently, it can be observed that positive effect of ultrasound treatment exceeded the effect of enzymatic maceration alone. Additionally, the correlation analysis of antioxidant activity and phenolic compounds content in raspberry juice showed a statistically significant correlation (r = 0.71). In the case of juice obtained from strawberries, the effect of ultrasound is also observed, when the treatment was performed without maceration a 16.0% increase in antioxidant activity was reached compared to ‘Control’. The combined treatment of sonication and maceration ‘US + E’ contributed to a 28.8% increase in its antioxidant activity. However, it can be noted that the juice obtained after maceration without sonication (‘Heated’) had similar antioxidant activity. Regardless of the method of pretreatment, blackcurrant juice had significantly higher antioxidant activity than the rest of the juices. However, a statistically significant increase in this parameter was observed under the effect of ultrasound treatment (‘US + E’) by 6.2% if compared to juice pressed after maceration (‘Heated + E’). 

Similar results were obtained in the study where three currant cultivars were tested: after 10 min of ultrasound mash treatment in a laboratory bath, at least a 10% increase in antioxidant capacity was reported [23]. The positive effect of ultrasonic treatment on juice processing was also previously reported. For example, there was an increase in DPPH free radical scavenging activity and total antioxidant capacity in grape juice samples sonicated for 90 min by 11.6% and 30.5%, respectively [27]. Studies conducted on strawberries also show that the use of heat or ultrasonic treatment of the mash can result in an increase in the antioxidant activity of strawberry juice compared to juice from untreated mash [34]. Such results are very similar to those highlighted in this study concerning blackcurrant fruit, but it is also worth noting that the effect on raspberry mash was much stronger. However, negative or no effect of ultrasound treatment on antioxidant capacity is reported. The juice obtained from *Berberis amurensis* Rupr. fruit mash after ultrasound treatment has the same antioxidant activity as the untreated ‘Control’ sample, while the antioxidant activity of juices made from the mash subjected to enzymatic maceration or heating was 55% higher [24]. 

## 4. Conclusions

Berries, in particular blackcurrants, but also raspberries and strawberries are a good source of polyphenolic compounds and ascorbic acid. It is also a good material used in the production of juices, but it is usually blended with other ingredients to make fruit drinks or other food products of a better taste profile. In addition to the quality of the raw material used, an important issue remains the degree of retention of these compounds in juices, on which the use of ultrasound can favor.

As it is shown in the following work, depending on the species, the effect of ultrasound treatment is different. A vivid example is the yield of juice pressing, where in the case of blackcurrant fruit, the use of ultrasound without enzymatic maceration was not enough to make it possible to produce juice. While for strawberry and raspberry fruit, the effect was comparable to the use of enzyme maceration. Further research on optimization may lead to the development of a method to completely replace the enzyme maceration with a method involving several minutes of ultrasound treatment. Such a solution can result not only in saving time but also in saving the energy required to heat the mash. What is more, such a solution also eliminates the need for dosing enzyme preparations and demanding logistics connected to it. It is noteworthy that none of the samples tested showed a negative effect of ultrasound on the total content of phenolic compounds. In raspberry juices pressed after ultrasound treatments, a significant increase in anthocyanins was observed in comparison to others juice samples. Higher extraction efficiency and shorter time of juice processing is also a way to increase antioxidant activity, which has been observed in cases of raspberry and blackcurrant. Antioxidant activities of these juices pressed after ultrasound treatment were higher compared to juices obtained without such treatment. In the case of strawberry juice, the temperature and time of processing were more important factors affecting phenolic compound content and antioxidant. The results of the experiment showed that, in order to better compare juice quality, separating the effect of temperature from the effect of mechanical and chemical action of ultrasound treatment is required. However, studies have shown that the impact of ultrasound may affect the share of individual anthocyanins, as was observed in the case of raspberry fruits. Ultrasonic interference in the anthocyanin structure may be important during the storage of raspberry juices, but this would require further research.

The use of ultrasound to treat fruit mash before pressing is a relatively new concept, and the number of publications on the subject is still insufficient, especially regarding the number of technological solutions employed in scientific research.

## Figures and Tables

**Table 1:** Soluble solids content [°Bx] in raspberry, strawberry, and blackcurrant juices obtained from differently treated mash.

Species	Sample–Mash Treatment				
	‘Control’	‘Heated’	‘US’	‘US + E’	‘Heated + E’
Raspberry	12.05 ± 0.26 a	12.04 ± 0.14 a	11.92 ± 0.37 a	11.89 ± 0.27 a	12.21 ± 0.24 a
Strawberry	7.25 ± 0.16 a	7.43 ± 0.05 ab	7.27 ± 0.19 a	7.54 ± 0.15 b	7.47 ± 0.08 ab
Blackcurrant	no juice	no juice	no juice	16.40 ± 0.09 a	16.50 ± 0.17 a

Means ± standard deviation; Means in the row marked with the same letter do not differ significantly (*p* = 0.05) according to Tukey’s test (n = 3).

**Table 2:** Contents of phenolic compounds (mg/100 mL) in raspberry juices obtained in different mash treatment.

Sample		Control	Heated	US	US + E	Heated + E
PHENOLIC ACIDS	Chlorogenic acid derivative A	0.72 ± 0.03 a	0.73 ± 0.05 a	0.72 ± 0.07 ab	0.68 ± 0.04 a	0.82 ± 0.09 b
	Chlorogenic acid derivative B	0.80 ± 0.23 a	0.91 ± 0.07 a	0.64 ± 0.29 a	0.68 ± 0.14 a	0.93 ± 0.11 a
	Ellagic acid	11.05 ± 0.89 a	9.98 ± 0.96 a	14.32 ± 0.81 b	13.92 ± 1.13 b	12.72 ± 1.30 ab
FLAVONOLS	Quercetin derivative	0.32 ± 0.07 a	0.39 ± 0.03 a	0.32 ± 0.08 a	0.33 ± 0.05 a	0.41 ± 0.02 a
	Quercetin-rutinoside	0.47 ± 0.09 a	0.54 ± 0.06 ab	0.61 ± 0.07 b	0.88 ± 0.07 c	0.66 ± 0.02 b
	Quercetin-galactoside	0.39 ± 0.30 a	0.64 ± 0.05 a	0.23 ± 0.36 a	0.39 ± 0.48 a	0.68 ± 0.12 a
	Quercetin-glucoside	2.26 ± 0.25 a	2.15 ± 0.07 a	2.53 ± 0.33 a	2.6 ± 0.29 a	2.14 ± 0.11 a
ANTHOCYANINS	Cyanidin 3-sophoroside	51.06 ± 4.97 a	50.58 ± 4.66 a	103.58 ± 1.34 b	97.66 ± 1.24 b	43.24 ± 7.6 a
	Cyanidin 3-glucosyl-rutinoside	12.79 ± 0.94 b	12.61 ± 1.14 b	0.57 ± 0.21 a	0.66 ± 0.18 a	13.01 ± 1.43 b
	Cyanidin-3-glucoside	11.92 ± 0.31 a	12.52 ± 0.68 a	16.80 ± 0.65 b	16.09 ± 0.29 b	11.73 ± 0.78 a
	Cyanidin 3-rutinoside	8.41 ± 0.80 b	8.45 ± 0.90 b	0.26 ± 0.40 a	0.57 ± 0.14 a	8.82 ± 1.56 b ^1^

^1^ Means ± standard deviation; Means in the row marked with the same letter do not differ significantly (*p* = 0.05) according to Tukey’s test (n = 3).

**Table 3:** Contents of phenolic compounds (mg/100 mL) in strawberry juices pressed after different mash treatment.

Sample		Control	Heated	US	US + E	Heated + E
FLAVANOLS	Procyanidin dimer B1	1.37 ± 0.05 a	1.64 ± 0.08 b	1.66 ± 0.27 b	1.80 ± 0.04 bc	1.90 ± 0.05 c
	Catechin	5.19 ± 0.43 abc	5.11 ± 0.17 ab	5.02 ± 0.17 a	5.52 ± 0.06 c	5.49 ± 0.09 bc
PHENOLIC ACIDS	Chlorogenic acid derivatives	15.26 ± 1.28 a	14.74 ± 0.71 a	15.94 ± 1.40 a	14.43 ± 0.69 a	14.64 ± 0.36 a
FLAVONOLS	Quercetin derivative A	0.17 ± 0.02 a	0.26 ± 0.04 b	0.27 ± 0.07 b	0.30 ± 0.01 b	0.28 ± 0.01 b
	Quercetin-3-rutinoside	1.61 ± 0.18 a	2.05 ± 0.21 b	1.60 ± 0.01 a	1.90 ± 0.24 ab	2.01 ± 0.20 b
	Quercetin-3-glucoside	0.11 ± 0.01 a	0.25 ± 0.32 a	0.11 ± 0.01 a	0.12 ± 0.01 a	0.11 ± 0.01 a
	Quercetin derivative B	0.27 ± 0.03 a	0.36 ± 0.02 bc	0.35 ± 0.05 b	0.40 ± 0.00 c	0.37 ± 0.02 bc
	Quercetin derivative C	0.11 ± 0.01 a	0.13 ± 0.01 b	0.13 ± 0.00 b	0.11 ± 0.00 a	0.11 ± 0.00 a
	Kaempferol-3-rutinoside	0.85 ± 0.05 a	0.95 ± 0.05 b	0.90 ± 0.03 ab	0.95 ± 0.06 b	0.96 ± 0.05 b
	Quercetin derivative D	0.24 ± 0.02 a	0.27 ± 0.01 b	0.27 ± 0.01 b	0.26 ± 0.01 ab	0.26 ± 0.01 ab
ANTHOCYANINS	Cyanidin-3-glucoside	2.03 ± 0.21 a	2.29 ± 0.06 ab	2.43 ± 0.28 b	2.35 ± 0.23 ab	2.31 ± 0.14 ab
	Pelargonidin-3-glucoside	32.82 ± 2.03 ab	34.34 ± 0.95 b	35.04 ± 0.95 b	31.62 ± 0.48 a	31.93 ± 1.66 a
	Pelargonidin-3-rutinoside	0.06 ± 0.05 a	0.08 ± 0.06 a	0.08 ± 0.01 a	0.06 ± 0.04 a	0.08 ± 0.01 a
	Cyanidin-3-malonylglucoside	0.25 ± 0.03 a	0.30 ± 0.01 bc	0.31 ± 0.01 c	0.28 ± 0.02 ab	0.28 ± 0.02 ab
	Pelargonidin-3-(6″-malonylglucoside)	4.51 ± 0.64 ab	5.04 ± 0.33 bc	5.18 ± 0.03 c	4.37 ± 0.06 a	4.42 ± 0.29 a ^1^

^1^ Means ± standard deviation; Means in the row marked with the same letter do not differ significantly (*p* = 0.05) according to Tukey’s test (n = 3).

**Table 4:** Contents of phenolic compounds (mg/100 mL) in blackcurrant juices obtained after different mash treatment.

Sample		US + E	Heated + E
PHENOLIC ACIDS	Chlorogenic acid derivatives A	1.92 ± 0.06 b	1.79 ± 0.07 a
	Chlorogenic acid derivatives B	5.18 ± 0.16 b	4.90 ± 0.07 a
FLAVONOLS	unidentified flavonol B	10.09 ± 0.46 a	10.30 ± 0.48 a
	Quercetin-3-rutinoside	3.10 ± 0.08 a	3.03 ± 0.24 a
	Quercetin-3-galactoside	0.43 ± 0.05 a	0.41 ± 0.05 a
	Quercetin-3-glucoside	6.94 ± 0.26 b	6.41 ± 0.21 a
	Quercetin	0.13 ± 0.01 a	0.13 ± 0.02 a
ANTHOCYANINS	Delphinidin-3-glucoside	28.08 ± 1.28 a	29.45 ± 0.71 b
	Delphinidin-3-rutinoside	97.56 ± 4.56 a	101.19 ± 2.36 a
	Cyanidin-3-glucoside	8.53 ± 0.32 a	9.02 ± 0.49 a
	Cyanidin-3-rutinoside	52.75 ± 2.20 a	54.93 ± 1.83 a
	Peonidin-3-glucoside	2.19 ± 0.11 a	2.29 ± 0.04 a
	Peonidin-3-rutinoside	0.75 ± 0.04 a	0.77 ± 0.01 a ^1^

^1^ Means ± standard deviation. Means in the row marked with the same letter do not differ significantly. (*p* = 0.05) according to Tukey’s test (n = 3).

**Table 5:** Contents of organic acids (mg/100 mL) in juices after different mash treatment.

Sample		Ascorbic Acid	Malic Acid	Citric Acid	Total
Raspberry	Control	24.57 ± 0.41 c	88.90 ± 5.00 b	2050 ± 58.00 a	2164 ± 56.00 a
	Heated	23.05 ± 1.07 bc	88.60 ± 7.80 b	2064 ± 59.00 a	2176 ± 63.00 a
	US	21.07 ± 1.47 ab	91.78 ± 6.30 b	1843 ± 201.00 a	1956 ± 196.00 a
	US + E	20.90 ± 0.86 ab	84.40 ± 9.80 a	1965 ± 95.00 a	2071 ± 86.00 a
	Heated + E	20.34 ± 0.71 a	82.10 ± 6.90 a	2054 ± 38.00 a	2160 ± 41.00 a
Strawberry	Control	33.12 ± 2.19 a	112.10 ± 27.70 ab	785.0 ± 71.00 a	931.0 ± 98.00 a
	Heated	29.86 ± 4.18 a	100.10 ± 16.20 ab	811.0 ± 39.00 a	941.0 ± 55.00 ab
	US	28.00 ± 3.34 a	90.30 ± 8.00 a	797.0 ± 12.00 a	915.0 ± 8.00 a
	US + E	29.58 ± 3.07 a	121.00 ± 12.50 b	888.0 ± 13.00 b	1038 ± 25.00 b
	Heated + E	28.47 ± 1.97 a	118.70 ± 19.50 ab	884.0 ± 33.00 b	1031 ± 56.00 b
Blackcurrant	Control	no juice	no juice	no juice	no juice
	Heated	no juice	no juice	no juice	no juice
	US	no juice	no juice	no juice	no juice
	US + E	129.00 ± 4.40 a	370.30 ± 30.40 a	4682 ± 97.00 a	5181 ± 110.0 a
	Heated + E	129.80 ± 1.90 a	401.80 ± 36.40 a	4718 ± 90.00 a	5250 ± 115.0 a ^1^

^1^ Means ± standard deviation. Means in the row marked with the same letter do not differ significantly (*p* = 0.05) according to Tukey’s test (n = 3).

## Data Availability

The original contributions presented in the study are included in the article, further inquiries can be directed to the corresponding author.
